# Sperm chromatin condensation defects and IVF outcomes: a retrospective cohort study

**DOI:** 10.7717/peerj.20749

**Published:** 2026-01-29

**Authors:** Wen Zhou, Shan Lu, Aiai Wang, Manbo Jiang, Sinan Li, Huanqun Zhou

**Affiliations:** 1 Department of Reproductive Medicine, The Second Affiliated Hospital of Guangzhou University of Chinese Medicine, Guangzhou, Guangdong, China; 2 You Zhaoling National Studio for the Inheritance of Famous Traditional Chinese Medicine, The Second Affiliated Hospital of Guangzhou University of Chinese Medicine, Guangzhou, Guangdong, China

**Keywords:** Sperm chromatin condensation defects, *in vitro* fertilization, Clinical pregnancy, Live birth, Embryonic development

## Abstract

**Background:**

The clinical impact of sperm chromatin condensation defects (SCCD) on *in vitro* fertilization (IVF) outcomes remains controversial. This study aimed to clarify the effects of SCCD on embryonic development, pregnancy, and neonatal outcomes in couples undergoing their first IVF cycle.

**Methods:**

This retrospective cohort study included 647 couples. Multivariable logistic regression was used to evaluate associations between SCCD (assessed by aniline blue staining) and clinical outcomes, with stratification and generalized additive models employed to identify effect modifiers and nonlinear relationships.

**Results:**

Elevated SCCD levels (≥30%) were correlated with abnormal conventional semen parameters and a reduced two-pronuclei (2PN) cleavage rate, whereas no significant associations were observed with spontaneous abortion, gestational age, birth weight, or neonatal sex distribution. After adjusting for key confounders, increasing SCCD levels remained independently associated with reduced clinical pregnancy (OR = 0.98, *P* = 0.01) and live birth rates (OR = 0.98, *P* = 0.02), and no significant effect modification by any subgroup variable was observed (all *P* for interaction > 0.05). Moreover, individuals with SCCD levels ≥30% showed a trend toward substantially reduced clinical pregnancy (OR = 0.64, *P* = 0.05) and live birth rates (OR = 0.65, *P* = 0.06). Nonlinear analysis further identified a significant risk threshold for live birth at 10.6% (OR = 0.86, *P* = 0.01), with risk plateauing until a declining trend emerged beyond 24.1%.

**Conclusions:**

This study provides evidence that SCCD is independently associated with reduced IVF success, supporting its assessment in pre-IVF evaluation.

## Introduction

Infertility, defined by the World Health Organization as the failure to achieve a clinical pregnancy after 12 months of unprotected intercourse (Rowe et al., 1993), affects approximately 15% of reproductive-age couples, with male factors contributing to 30% to 50% of cases (Minhas et al., 2025). In 2021, an estimated 55 million men were affected by infertility globally, with male infertility rates projected to surpass female rates by 2040 (Liang et al., 2025). Conventional semen parameters remain the cornerstone of male fertility assessment, yet it fails to identify abnormalities in 30–40% of infertile men (Minhas et al., 2025), suggesting the need for extended examinations.

Sperm chromatin condensation defects (SCCD) arise from the abnormal substitution of histones and protamines, leading to incomplete chromatin compaction, which may affect sperm function (Torres-Flores & Hernández-Hernández, 2020). The WHO Laboratory Manual includes the assessment of SCCD within its section on extended examinations, underscoring its relevance in the comprehensive evaluation of male infertility (World Health Organization, 2021). Several studies suggest that these abnormalities may be associated with decreased fertilization rates and abnormal embryo developmental dynamics, through mechanisms impairing paternal genome decondensation, epigenetic reprogramming, and oxidative stress responses (Yamaguchi et al., 2018; Wyck et al., 2018; Tavalaee, Razavi & Nasr-Esfahani, 2009). Various studies indicate that these defects may lower fertilization and clinical pregnancy rates in *in vitro* fertilization (IVF) (Gu et al., 2009; Hammadeh et al., 1998), impair blastocyst formation, pregnancy, and live birth rates in intracytoplasmic sperm injection (ICSI) (Ribas-Maynou et al., 2023), and contribute to decreased success rates of intrauterine insemination (IUI), particularly in conjunction with abnormal semen parameters (Lazaros et al., 2011). Conversely, other evidence indicates that although chromatin abnormalities correlate with sperm morphology and motility (Angelopoulos et al., 1998), they do not significantly influence fertilization outcomes in IVF/ICSI (Angelopoulos et al., 1998; Jumeau et al., 2022), and these effects on embryo developmental dynamics do not result in differences in pregnancy rates (Jumeau et al., 2022). Consequently, the impact of SCCD on clinical IVF outcomes remains inconsistent across studies, potentially arising from methodological heterogeneity in SCCD thresholds, assessment methods, and population characteristics. Thus, critical questions remain: whether SCCD is an independent risk factor for IVF outcomes after rigorous confounder adjustment, and crucially, the nature of this association (*e.g*., linear or threshold effect) and how consistent it is across different patient subpopulations.

To address these questions, we conducted a retrospective cohort study of 647 first IVF cycles. Our analysis aimed to (1) examine the independent association between SCCD and embryo development, pregnancy, and neonatal outcomes with multivariable models; (2) characterize the dose-response relationship by testing for threshold effects; and (3) assess the consistency of these associations across patient subgroups. This study provides a robust evidence base to inform the potential clinical application of SCCD assessment.

## Materials and Methods

### Study population

**Figure 1:**
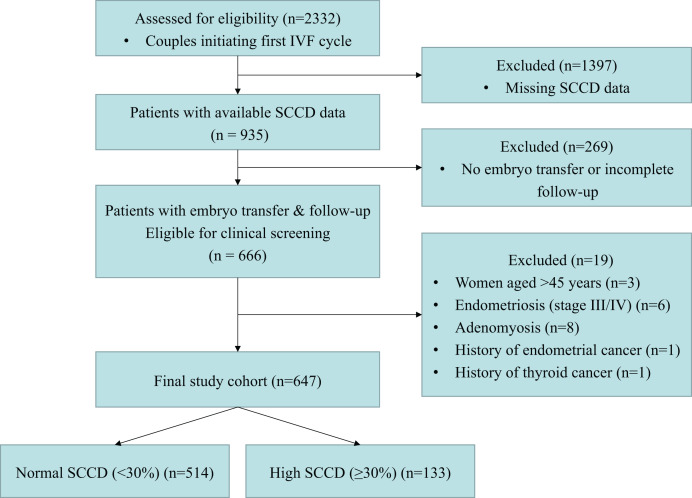
Study flow diagram. The flowchart illustrates participant selection. The final cohort was stratified by SCCD level using a 30% threshold (Normal: SCCD < 30%; High: SCCD ≥ 30%). **Abbreviations:** SCCD, sperm chromatin condensation defect; IVF, *in vitro* fertilization.

This retrospective cohort study screened a consecutive series of 2,332 infertile couples initiating their first IVF cycle at the Reproductive Medicine Department of Guangdong Provincial Hospital of Chinese Medicine (May 2017–June 2024). The first cycle was selected to minimize potential confounders from prior IVF treatments and to ensure statistical independence. To ensure data robustness, 1,397 cases with missing SCCD assessment and 269 cases with no embryo transfer or incomplete pregnancy follow-up were excluded upfront. From the resulting 666 cases, 19 were further excluded based on clinical criteria: female age > 45 years or BMI ≥ 36 kg/m^2^; stage III/IV endometriosis, adenomyosis, or other specified gynecological pathologies; male age ≥ 55 years or severe systemic disease; and general IVF contraindications. Based on the SCCD levels, participants were classified into two groups: the normal SCCD group (<30%, *n* = 514) and the high SCCD group (≥30%, *n* = 133) (Fig. 1). This 30% threshold was established in accordance with published literature (Auger et al., 1990; Hammadeh et al., 2001), the manufacturer’s instructions for the staining kit, and prevailing clinical laboratory standards in China (Tang, Zhang & Lu, 2019). This study was approved by the Ethics Committee of Guangdong Provincial Hospital of Chinese Medicine (approval number: ZE2025-235). Under the Chinese Ethical Review Measures for Life Science and Medical Research Involving Humans, the study met all criteria for a waiver of informed consent, being a retrospective analysis of anonymized data that posed minimal risk and where re-contacting participants was impracticable.

### SCCD assessment

Sperm chromatin condensation was evaluated using aniline blue staining based on the WHO laboratory manual (World Health Organization, 2021) with modifications from Wong et al. (2008). The assessment was performed using the Sperm Nuclear Protein Staining Kit (Boride Biotechnology Co., Ltd., Shenzhen, China) following the manufacturer’s protocol. Following semen liquefaction, sperm concentration was adjusted to 20–40 × 10^6^/mL. A 5 μl aliquot was smeared within a defined slide area, fixed with 4% formalin solution for 2 min, stained with aniline blue solution (in 4% glacial acetic acid, pH 3.5) for 5 min, treated with 90% ethanol for 5 min, and counterstained with 0.5% eosin for 5 min. After air-drying, slides were examined under oil immersion at 1,000× magnification using bright-field microscopy. With this staining method, sperm with normal chromatin condensation (rich in protamines) appear reddish-pink, while those with abnormal condensation (containing lysine-rich histones) stain purplish-blue. All assessments were performed in a blinded manner by one of two experienced technologists following standardized protocols. A minimum of 200 spermatozoa per sample were counted twice, and the mean value was adopted if the difference between duplicates was within the allowable margin of error defined by the 95% confidence interval in the WHO laboratory manual (World Health Organization, 2021); otherwise, a recount was performed. Internal quality control samples were included in each batch, and system performance was monitored using Levey-Jennings control charts throughout the study. A representative micrograph is shown in Fig. S1.

### Ovarian stimulation

Ovarian stimulation protocols were personalized based on female age, endocrine profiles, and ovarian reserve. The final oocyte maturation was triggered with either human chorionic gonadotropin (hCG) or a gonadotropin-releasing hormone agonist (GnRH agonist). The following protocols were utilized: (1) Long and ultra-long GnRH agonist protocols: These protocols employed GnRH agonist for pituitary down-regulation. Gonadotropin (Gn) preparations (including recombinant or urinary-derived FSH, LH, and hMG) were initiated at 75–300 IU/day after achieving down-regulation. These protocols were applied to a broad spectrum of patients undergoing assisted reproduction, though they were used with caution in those with poor ovarian reserve. (2) GnRH antagonist protocols: This protocols used GnRH antagonists to suppress premature LH surges. There were particularly indicated for patients with polycystic ovary syndrome (PCOS) or a high ovarian response. To mitigate the risk of ovarian hyperstimulation syndrome (OHSS), a GnRH agonist trigger was often employed. (3) Micro-stimulation protocol: This protocol involved clomiphene citrate, letrozole, or low-dose Gn. It was specifically suitable for patients with diminished ovarian reserve. The starting dose of Gn was individualized based on female age, antral follicle count, and BMI. Typical starting doses stratified by age were: <30 years: 100–112.5 IU/day; 30–35 years: 112.5–150 IU/day; and ≥ 35 years: 150–225 IU/day. Follicular development was monitored *via* ultrasonography and serum hormone measurements. The timing of the ovulation trigger was determined by lead follicular size, serum estradiol and LH levels, and the specific stimulation protocol. Oocyte retrieval was performed 34–38 h after trigger administration.

### Fertilization and embryo culture

The oocyte-cumulus complexes were pre-cultured for 4–6 h in a 37 °C incubator with 6% CO_2_. The spermatozoa were processed by discontinuous density gradient centrifugation to select a population of sperm with high motility. Subsequently, sperm and oocytes were co-incubated for 16–18 h, after which the fertilization status (presence of two pronuclei) was assessed on Day 1. Embryo development was then evaluated on Days 2 and 3, assessing parameters such as blastomere count, uniformity, fragmentation, multinucleation, and vacuolation. Blastocyst culture was selectively performed based on specific clinical indications, including a favorable prognosis (young age, ample oocytes and high-quality embryos), a history of recurrent implantation failure, or as a rescue strategy for suboptimal cleavage-stage embryos. Supernumerary embryos were cryopreserved using vitrification. Finally, the rates of fertilization, usable embryos, high-quality embryos, and blastocyst formation were calculated.

### Embryo transfer

Grade 1–3 cleavage-stage (Day 3) embryos or viable blastocysts (Day 5/6) were selected for transfer, with a maximum of two embryos per cycle. Elective single embryo transfer (eSET) was strongly recommended for patients under 38 years with optimal endometrial conditions and high-quality blastocysts to minimize multiple pregnancies, and was mandatory in cases of uterine factors or maternal conditions unsuitable for twin gestation. Fresh embryo transfer was cancelled in cases of elevated risk for ovarian hyperstimulation syndrome (OHSS), premature progesterone elevation, suboptimal endometrium, or following specific stimulation protocols (*e.g*., clomiphene-based cycles). Luteal phase support was initiated on the day of oocyte retrieval. For hCG-triggered cycles, support consisted of progesterone, with low-dose hCG supplementation considered for patients with a low ovarian response. For GnRH agonist-triggered cycles, the protocol was stratified based on the trigger-day estradiol (E2) level: for E2 < 14,680 pmol/L, a low-dose hCG (1,500–2,000 IU) bolus was administered on retrieval day, followed by standard progesterone; for E2 ≥ 14,680 pmol/L, intensive support with progesterone and estrogen was used, avoiding hCG. Luteal support was typically maintained until 10 weeks of gestation. For frozen-thawed embryo transfer (FET), endometrial preparation was achieved through natural, hormone replacement therapy (HRT), or ovulation induction cycles, tailored to the patient’s ovulatory status and endocrine profile.

### Pregnancy confirmation and follow-up

Serum β-human chorionic gonadotropin (β-hCG) testing was performed approximately 14 days after embryo transfer to determine biochemical pregnancy. Patients with a positive result continued luteal support and were monitored for outcome. Clinical pregnancy was confirmed around 30 days post-transfer by transvaginal ultrasonography to determine the pregnancy location, number of gestational sacs, and fetal cardiac activity. In cases suggestive of multiple gestation, a follow-up scan was arranged one week later to confirm, and the associated risks were thoroughly discussed, with multifetal pregnancy reduction offered as a clinical option when appropriate. A subsequent ultrasound was performed around 50 days post-transfer to confirm ongoing fetal viability. Fetal anatomy and development were further assessed during the second trimester (approximately 16–20 weeks of gestation). Finally, a structured telephone follow-up was conducted at 10 months post-transfer to ascertain delivery mode, gestational age at delivery, and neonatal outcomes, including birth weight, sex, and health status.

### Statistical analyses

Data were analyzed using EmpowerStats (version 4.2) and R software (version 3.4.3; R Core Team, 2018). Continuous variables were presented as mean ± standard deviation (SD) or median (interquartile range, IQR) based on normality assessment using the Shapiro-Wilk test, with group comparisons performed using t-tests or Mann-Whitney U tests. Categorical variables were expressed as counts (percentages) and compared using chi-square or Fisher’s exact tests. Sample size adequacy was confirmed by the Events Per Variable (EPV) rule; values for live birth (19.1) and clinical pregnancy (22.9) exceeded the recommended threshold of 10 (Austin, Allignol & Fine, 2017). Multivariable logistic regression models adjusted for confounders selected based on: (1) >10% change in the SCCD regression coefficient upon variable inclusion or exclusion; (2) association with the outcome at *P* < 0.1; (3) established clinical relevance; and (4) variance inflation factors (VIF) <5. Additional analyses included generalized additive models (GAM) for nonlinearity, segmented logistic regression for threshold effects, and subgroup analyses with interaction tests. Results are reported as odds ratios (OR) or coefficients (β) with 95% confidence intervals (CI). All tests were two-tailed with *P* < 0.05 considered statistically significant.

## Results

### Demographic and clinical characteristics

As detailed in Table 1, the median age of females was lower in the high SCCD group than in the normal SCCD group (31.00 [29.00–35.00] *vs*. 33.00 [30.00–35.00] years, *P* = 0.04). The high SCCD group had a significantly higher proportion of male factor infertility (25.56% *vs*. 14.40%) and a correspondingly lower proportion of female factor infertility (60.15% *vs*. 71.60%) (*P* < 0.01). Furthermore, primary infertility was significantly more prevalent in the high SCCD group (60.90% *vs*. 48.05%, *P* = 0.01). No significant intergroup differences were observed for other parameters, including male age, female BMI, reproductive hormone levels, ovarian reserve indicators, and ovarian stimulation characteristics (all *P* > 0.05).

**Table 1:** Baseline characteristics of the study population by SCCD status.

	Total (*n* = 647) (1.30–78.10)	SCCD < 30% (*n* = 514) (1.30–29.90)	SCCD ≥ 30% (*n* = 133) (30.00–78.10)	*P* value
SCCD (%)	18.50 (12.00–27.60)	15.60 (11.00–21.75)	36.10 (32.40–42.00)	<0.001
Female age (years)	32.00 (30.00–35.00)	33.00 (30.00–35.00)	31.00 (29.00–35.00)	0.04
Male age (years)	34.00 (31.00–37.00)	34.00 (31.00–37.00)	33.00 (30.00–37.00)	0.57
Female BMI (kg/m^2^)	21.76 (19.85–24.02)	21.78 (19.85–24.01)	21.45 (19.85–24.16)	0.93
Infertility duration (years)	2.00 (1.00–4.00)	2.00 (1.00–4.00)	3.00 (1.50–4.00)	0.31
Baseline FSH (mIU/ml)	6.74 (5.76–7.99)	6.72 (5.76–8.00)	6.75 (5.79–7.91)	0.97
Baseline LH (mIU/mL)	5.61 (4.44–7.47)	5.62 (4.44–7.44)	5.57 (4.30–7.54)	0.74
Baseline E2 (pmol/L)	148.10 (113.85–192.85)	148.83 (116.10–194.38)	145.40 (106.22–183.00)	0.19
AMH (ng/mL)	3.12 (1.70–4.72)	3.05 (1.66–4.74)	3.48 (1.84–4.67)	0.21
AFC	15.00 (10.00–23.00)	15.00 (9.25–22.75)	16.00 (10.00–23.00)	0.73
Gn starting dose (IU)	162.50 (137.50–225.00)	156.25 (137.50–225.00)	187.50 (125.00–225.00)	0.93
Gn duration (days)	11.00 (10.00–12.00)	11.00 (10.00–12.00)	11.00 (10.00–12.00)	0.83
Total Gn (IU)	1,887.50 (1,443.75–2,412.50)	1,875.00 (1,450.00–2,400.00)	1,925.00 (1,437.50–2,425.00)	0.79
Trigger day LH (mIU/mL)	2.33 (1.51–3.62)	2.34 (1.55–3.73)	2.21 (1.39–3.35)	0.33
Trigger day E2 (pmol/L)	11,104.00 (7,567.75–17,277.25)	11,196.00 (7,556.00–17,335.00)	11,010.00 (7,799.00–16,933.00)	0.96
Trigger day P (nmol/L)	2.26 (1.53–3.42)	2.27 (1.53–3.42)	2.26 (1.52–3.45)	0.84
Endometrial thickness (mm)	10.00 (8.50–12.00)	10.00 (8.30–12.00)	10.00 (9.00–11.70)	0.57
**Stimulation protocol**				0.32
Antagonist	351 (54.33%)	274 (53.41%)	77 (57.89%)	
Long	116 (17.96%)	87 (16.96%)	29 (21.80%)	
PPOS	68 (10.53%)	60 (11.70%)	8 (6.02%)	
Mild stimulation	37 (5.73%)	32 (6.24%)	5 (3.76%)	
Ultra long	36 (5.57%)	30 (5.85%)	6 (4.51%)	
Modified long	36 (5.57%)	28 (5.46%)	8 (6.02%)	
Gentle stimulation	2 (0.31%)	2 (0.39%)	0 (0.00%)	
**Infertility factors**				<0.01
Female	448 (69.24%)	368 (71.60%)	80 (60.15%)	
Male	108 (16.69%)	74 (14.40%)	34 (25.56%)	
Combined	72 (11.13%)	54 (10.51%)	18 (13.53%)	
Unexplained	19 (2.94%)	18 (3.50%)	1 (0.75%)	
**Infertility types**				0.01
Primary	328 (50.70%)	247 (48.05%)	81 (60.90%)	
Secondary	319 (49.30%)	267 (51.95%)	52 (39.10%)	

**Note:**

Continuous non-normally distributed variables are summarized as median (interquartile range) and compared with the Mann-Whitney U test. Categorical variables are presented as number (percentage) and compared with the Chi-square test. Abbreviations: SCCD, sperm chromatin condensation defect s; BMI, body mass index; FSH, follicle-stimulating hormone; LH, luteinizing hormone; E2, estradiol; AMH, anti-Müllerian hormone; AFC, antral follicle count; Gn, gonadotropin; P, progesterone.

### Semen and embryo development parameters

Comparative analysis revealed several significant associations between elevated SCCD and semen parameters (Table 2). The high SCCD group exhibited markedly reduced sperm concentration (40.00 [30.00–53.00] *vs*. 47.00 [35.00–67.00] × 10^6^ mL^−1^, *P* < 0.001), normal morphology rate (3.50 [2.00–5.00] *vs*. 4.50 [3.00–6.27]%, *P* < 0.001), and optimized sperm concentration (43.00 [30.00–55.00] *vs*. 48.00 [36.00–63.00] × 10^6^ mL^−1^, *P* < 0.01). A lower percentage of progressively motile sperm was also observed (38.00 [30.00–42.00] *vs*. 39.00 [33.00–45.00]%, *P* = 0.02). Regarding embryological outcomes, the high SCCD group was associated with a significantly lower 2-pronuclei (2PN) cleavage rate (98.49% *vs*. 99.47%, *P* < 0.001). Conversely, no significant differences were found between the groups in semen volume, optimized progressive motility, normal fertilization rate (2PN), usable embryo rate, high-quality embryo rate, and blastocyst formation rate (all *P* > 0.05).

**Table 2:** Semen parameters and embryo development outcomes by SCCD status.

	Total (*n* = 647) (1.30–78.10)	SCCD < 30% (*n* = 514) (1.30–29.90)	SCCD ≥ 30% (*n* = 133) (30.00–78.10)	*P* value
Semen volume (mL)	2.00 (2.00–3.00)	2.00 (2.00–3.00)	2.50 (2.00–3.00)	0.30
Sperm concentration (10^6^ mL^−1^)	45.00 (34.00–63.00)	47.00 (35.00–67.00)	40.00 (30.00–53.00)	<0.001
Progressive sperm motility (%)	39.00 (33.00–45.00)	39.00 (33.00–45.00)	38.00 (30.00–42.00)	0.02
Optimized sperm concentration (10^6^ mL^−1^)	46.00 (35.00–60.00)	48.00 (36.00–63.00)	43.00 (30.00–55.00)	<0.01
Optimized progressive sperm motility (%)	83.00 (80.00–85.00)	83.00 (80.00–85.00)	82.00 (79.00–85.00)	0.49
Normal sperm morphology (%)	4.10 (3.00–6.00)	4.50 (3.00–6.27)	3.50 (2.00–5.00)	<0.001
Oocytes retrieved	7,870	6,236	1,634	
Normally fertilized (2PN) rate (%)	5,240 (66.58%)	4,180 (67.03%)	1,060 (64.87%)	0.10
2PN cleavage-stage embryos rate (%)	5,202 (99.27%)	4,158 (99.47%)	1,044 (98.49%)	<0.001
1PN cleavage-stage embryos	412	326	86	
Usable embryos rate (%)	4,723 (84.13%)	3,788 (84.48%)	935 (82.74%)	0.15
High-quality embryos rate (%)	1,860 (35.76%)	1,487 (35.76%)	373 (35.73%)	0.98
Number of blastocyst cultures	3,177	2,544	633	
Blastocyst formation rate (%)	2,276 (71.64%)	1,807 (71.03%)	469 (74.09%)	0.13

**Note:**

Continuous non-normally distributed variables are presented as median (interquartile range) and compared with the Mann-Whitney U test. Categorical outcomes are presented as number (percentage) and compared with the Chi-square test. Abbreviations: SCCD, sperm chromatin condensation defects; 2PN, two pronuclei; 1PN, one pronuclei.

### Clinical and neonatal outcomes

Comparative analysis revealed no significant differences in the primary clinical or neonatal outcomes between the high and normal SCCD groups (Table 3). Key clinical metrics, including embryo transfer parameters, implantation rate (37.85% *vs*. 42.49%, *P* = 0.26), clinical pregnancy rate (47.37% *vs*. 50.00%, *P* = 0.59), live birth rate (38.35% *vs*. 42.02%, *P* = 0.44), and spontaneous abortion rate (17.46% *vs*. 14.40%, *P* = 0.54), were all comparable. Similarly, neonatal outcomes showed no significant intergroup differences, as evidenced by gestational age at birth (39.00 [37.75–39.50] *vs*. 38.50 [37.50–39.50] weeks, *P* = 0.39), neonatal birth weight (3.10 [2.80–3.36] *vs*. 3.10 [2.62–3.44] kg, *P* = 0.67), incidence of congenital abnormalities (0.00% *vs*. 1.39%, *P* = 0.40), and neonatal sex ratio (male: 56.60% *vs*. 55.08%, *P* = 0.84).

**Table 3:** Clinical and neonatal outcomes by SCCD status.

	Total (*n* = 647) (1.30–78.10)	SCCD < 30 (*n* = 514) (1.30–29.90)	SCCD ≥ 30 (*n* = 133) (30.00–78.10)	*P*-value
**Transfer types**				0.20
Fresh	251 (38.79%)	193 (37.55%)	58 (43.61%)	
Frozen	396 (61.21%)	321 (62.45%)	75 (56.39%)	
**Embryos transferred**				0.74
1	425 (65.69%)	336 (65.37%)	89 (66.92%)	
2	222 (34.31%)	178 (34.63%)	44 (33.08%)	
**High-quality embryos transferred**				0.30
0	112 (17.31%)	89 (17.32%)	23 (17.29%)	
1	451 (69.71%)	353 (68.68%)	98 (73.68%)	
2	84 (12.98%)	72 (14.01%)	12 (9.02%)	
**Implanted embryos**				0.21
0	327 (50.54%)	257 (50.00%)	70 (52.63%)	
1	279 (43.12%)	220 (42.80%)	59 (44.36%)	
2	41 (6.34%)	37 (7.20%)	4 (3.01%)	
**Implantation rate**	361/869 (41.54%)	294/692 (42.49%)	67/177 (37.85%)	0.26
**Clinical pregnancy**				0.59
No	327 (50.54%)	257 (50.00%)	70 (52.63%)	
Yes	320 (49.46%)	257 (50.00%)	63 (47.37%)	
**Spontaneous abortion**				0.54
No	272 (85.00%)	220 (85.60%)	52 (82.54%)	
Yes	48 (15.00%)	37 (14.40%)	11 (17.46%)	
**Gestational age (weeks)**				
<37	35 (13.06%)	32 (14.75%)	3 (5.88%)	0.20
>=37, <41	224 (83.58%)	178 (82.03%)	46 (90.20%)	
>=41	9 (3.36%)	7 (3.23%)	2 (3.92%)	
**Live birth**				0.44
No	380 (58.73%)	298 (57.98%)	82 (61.65%)	
Yes	267 (41.27%)	216 (42.02%)	51 (38.35%)	
**Neonatal anomalies**				0.40
No	264 (98.88%)	213 (98.61%)	51 (100.00%)	
Yes	3 (1.12%)	3 (1.39%)	0 (0.00%)	
**Neonatal sex**				0.84
Male	154 (54.61%)	130 (55.08%)	30 (56.60%)	
Female	128 (45.39%)	106 (44.92%)	23 (43.40%)	
**Gestational age (weeks)**	38.50 (37.50–39.50)	38.50 (37.50–39.50)	39.00 (37.75–39.50)	0.39
**Birth weight (kg)**	3.10 (2.65–3.40)	3.10 (2.62–3.44)	3.10 (2.80–3.36)	0.67

**Note:**

Continuous non-normally distributed variables are presented as median (interquartile range) and compared with the Mann-Whitney U test. Categorical variables are presented as number (percentage) and compared with the Chi-square test. Abbreviations: SCCD, sperm chromatin condensation defects.

### Multivariable analysis of IVF outcomes

Multivariable regression analyses, adjusted for outcome-specific potential confounders (Table 4), revealed that higher SCCD was significantly associated with lower probabilities of clinical pregnancy and live birth. When analyzed as a continuous variable, each unit increase in SCCD was associated with a 2% reduction in the likelihood of achieving clinical pregnancy (adjusted OR = 0.98, 95% CI [0.96–0.99], *P* = 0.01) and live birth (adjusted OR = 0.98, 95% CI [0.96–1.00], *P* = 0.02). In the categorical analysis, compared to the normal SCCD group (SCCD <30%), the high SCCD group (SCCD ≥30%) showed a trend toward a 36% lower probability of clinical pregnancy (adjusted OR = 0.64, 95% CI [0.41–1.00], *P* = 0.05) and a 35% lower probability of live birth (adjusted OR = 0.65, 95% CI [0.41–1.02], *P* = 0.06). The robustness of these primary findings was supported by sensitivity analyses using simplified covariate sets (Table S1). No significant associations were observed between SCCD and other examined outcomes, including spontaneous abortion, gestational age, birth weight, or neonatal sex, in either crude or adjusted models (all *P* > 0.05).

**Table 4:** Multivariable regression analyses of associations between SCCD and clinical and neonatal outcomes.

	Crude	Adjusted
**Clinical pregnancy** ^*^		
Continuous	0.99 (0.98, 1.01) 0.24	0.98 (0.96, 0.99) 0.01
<30	Reference	Reference
≥30	0.90 (0.61, 1.32) 0.59	0.64 (0.41, 1.00) 0.05
**Spontaneous abortion** ^*^		
Continuous	1.02 (0.99, 1.04) 0.21	1.01 (0.98, 1.04) 0.61
<30	Reference	Reference
≥30	1.26 (0.60, 2.63) 0.54	1.14 (0.50, 2.58) 0.76
**Live birth** ^*^		
Continuous	0.99 (0.98, 1.00) 0.11	0.98 (0.96, 1.00) 0.02
<30	Reference	Reference
≥30	0.86 (0.58, 1.27) 0.44	0.65 (0.41, 1.02) 0.06
**Gestational age (weeks)** ^**#**^		
Continuous	0.01 (−0.01, 0.03) 0.22	0.01 (−0.01, 0.03) 0.39
<30	Reference	Reference
≥30	0.33 (−0.22, 0.88) 0.24	0.36 (−0.21, 0.93) 0.22
**Birth weight (kg)** ^**#**^		
Continuous	−0.00 (−0.03, 0.02) 0.79	−0.01 (−0.03, 0.01) 0.32
<30	Reference	Reference
≥30	−0.10 (−0.72, 0.52) 0.75	−0.25 (−0.88, 0.38) 0.44
**Neonatal sex (male)** ^*^		
Continuous	0.99 (0.97, 1.01) 0.42	0.98 (0.96, 1.01) 0.12
<30	Reference	Reference
≥30	1.06 (0.58, 1.94) 0.84	0.94 (0.48, 1.86) 0.87

**Note:**

Data are presented as OR (95% CI) *P*-value for logistic regression models (*) or β (95% CI) *P*-value for linear models (^#^). The SCCD categorical variable was dichotomized at the 30% threshold. All models were adjusted for outcome-specific confounders listed below. Clinical pregnancy and live birth: Female age, male age, female BMI, infertility factors, AMH, baseline FSH, endometrial thickness, stimulation protocol, Gn starting dose, Gn duration, trigger day E2, embryos transferred, high-quality embryos transferred, normal sperm morphology. Spontaneous abortion: Female age, female BMI, AMH, endometrial thickness, high-quality embryos transferred. Gestational age: Female age, female BMI, infertility factors, normal sperm morphology, optimized progressive sperm motility, baseline LH, AMH, Gn duration, transfer types, high-quality embryos transferred, embryos transferred. Neonatal sex (male): Progressive sperm motility, total sperm motility, baseline FSH, baseline LH, infertility factors, AFC, Gn duration, implanted embryos, high-quality embryos transferred. Birth weight: Female age, female BMI, infertility types, infertility factors, endometrial thickness, trigger day E2, transfer types, high-quality embryos transferred, implanted embryos. Abbreviations: SCCD, sperm chromatin condensation defects; OR, odds ratio; CI, confidence interval; BMI, body mass index; AMH, anti-Müllerian hormone; FSH, follicle-stimulating hormone; Gn, gonadotropin; E2, estradiol.

### Stratified analysis and interaction tests

**Figure 2:**
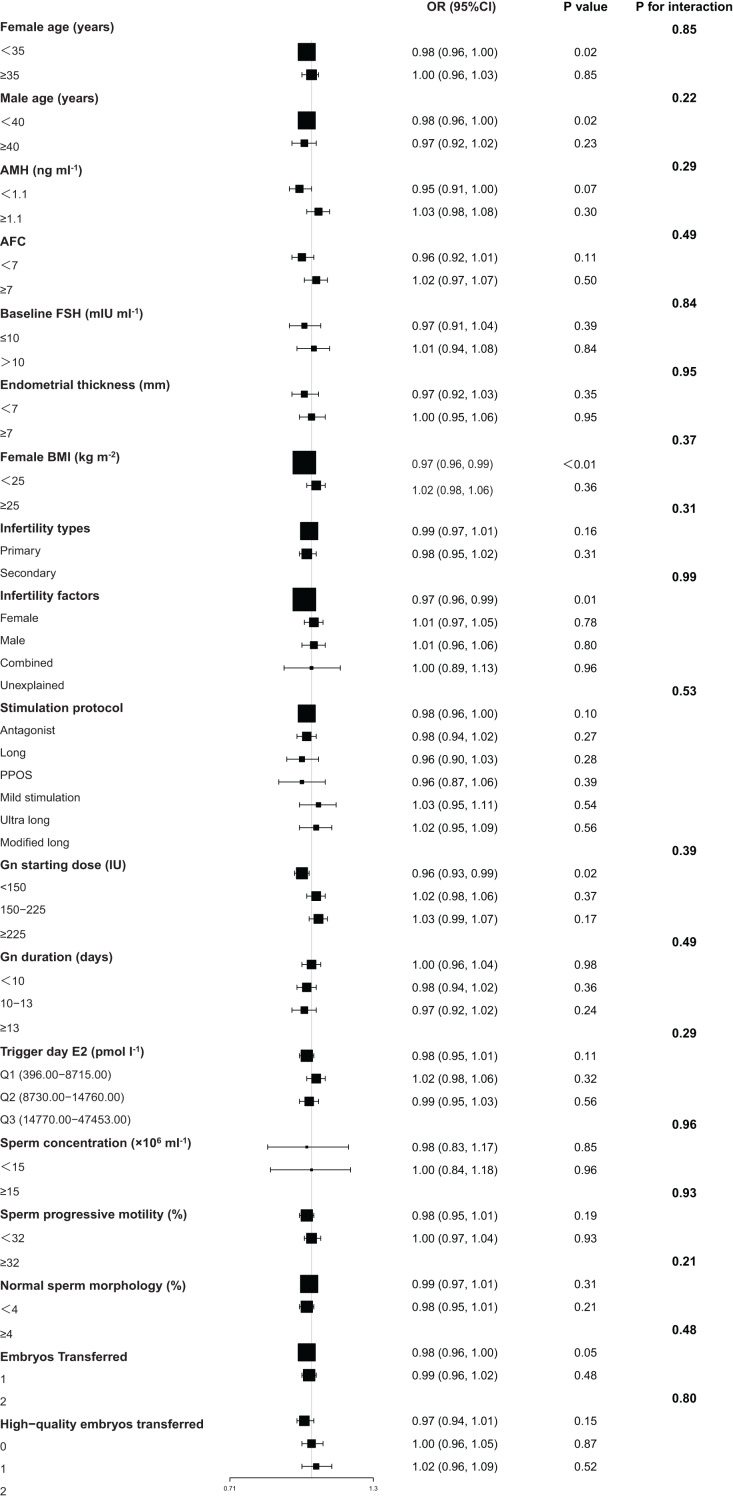
Subgroup analyses of the association between SCCD and clinical pregnancy. Results of multivariable logistic regression are displayed as a forest plot. All models were adjusted for female age, male age, BMI, infertility factors, AMH, baseline FSH, endometrial thickness, stimulation protocol, Gn starting dose, Gn duration, trigger day E2, embryos transferred, high-quality embryos transferred, and normal sperm morphology (excluding the stratification variable). Data are presented as OR with 95% CI and *P* values. *P* values for interaction were derived from likelihood ratio tests. Abbreviations: SCCD, sperm chromatin condensation defect; OR, odds ratio; CI, confidence interval; BMI, body mass index; AMH, anti-Müllerian hormone; FSH, follicle-stimulating hormone; Gn, gonadotropin; E2, estradiol.

**Figure 3:**
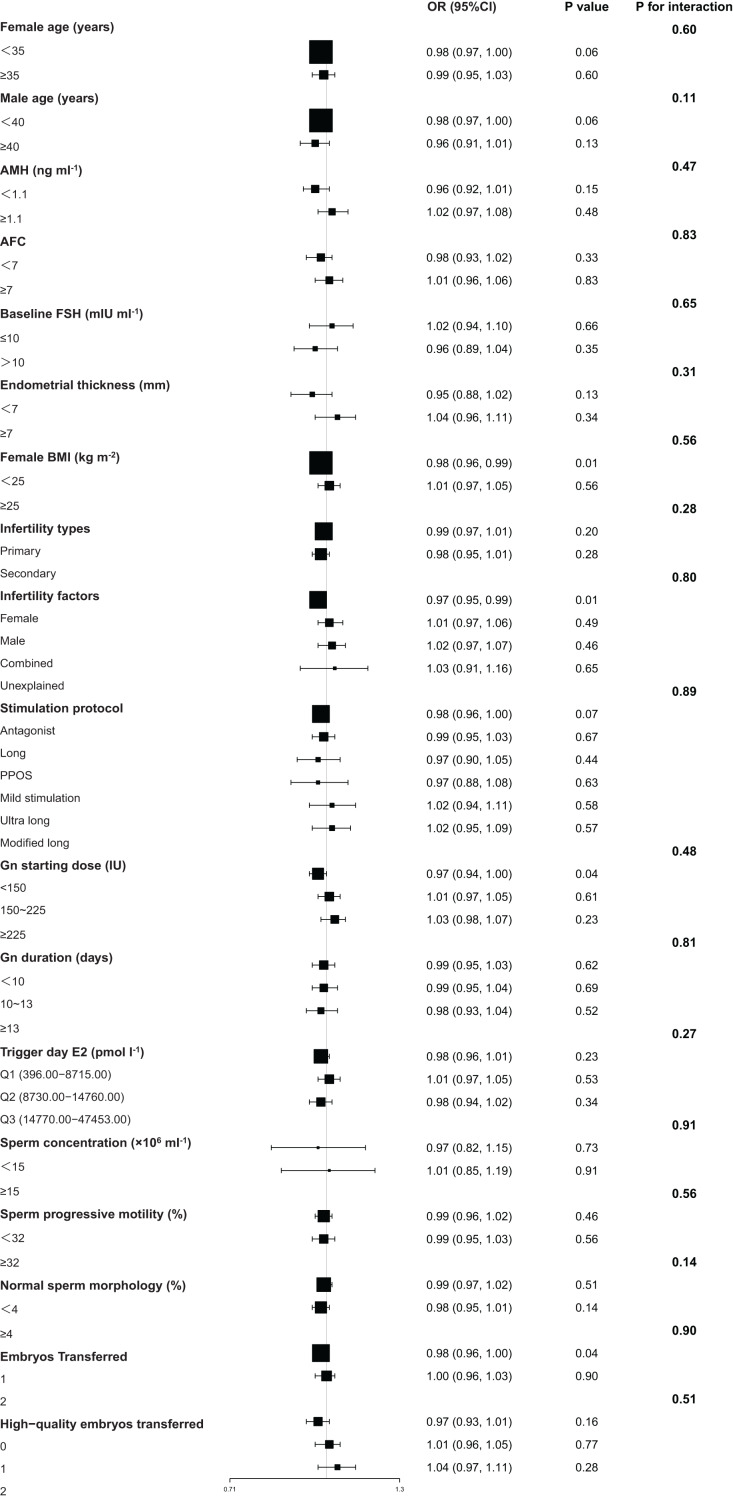
Subgroup analyses of the association between SCCD and live birth. Results of multivariable logistic regression are displayed as a forest plot. All models were adjusted for female age, male age, BMI, infertility factors, AMH, baseline FSH, endometrial thickness, stimulation protocol, Gn starting dose, Gn duration, trigger day E2, embryos transferred, high-quality embryos transferred, and normal sperm morphology (excluding the stratification variable). Data are presented as OR with 95% CI and *P* values. *P*-values for interaction were derived from likelihood ratio tests. Abbreviations: SCCD, sperm chromatin condensation defects; OR, odds ratio; CI, confidence interval; BMI, body mass index; AMH, anti-Müllerian hormone; FSH, follicle-stimulating hormone; Gn, gonadotropin; E2, estradiol.

Elevated SCCD was associated with lower probabilities of clinical pregnancy and live birth within specific patient subgroups (Figs. 2 and 3). For clinical pregnancy, a significant negative association was identified in younger women (<35 years, OR = 0.98, 95% CI [0.96–1.00], *P* = 0.02), women with normal BMI (<25 kg/m^2^, OR = 0.97, 95% CI [0.96–0.99], *P* < 0.01), cases of female factor infertility (OR = 0.97, 95% CI [0.96–0.99], *P* = 0.01), younger men (<40 years, OR = 0.98, 95% CI [0.96–1.00], *P* = 0.02), and cycles with a low Gn starting dose (<150 IU, OR = 0.96, 95% CI [0.93–0.99], *P* = 0.02). Additionally, a marginal negative association was observed in cycles with single embryo transfer (OR = 0.98, 95% CI [0.96–1.00], *P* = 0.05) (Fig. 2). For live birth, a significant negative association was found in women with normal BMI (OR = 0.98, 95% CI [0.96–0.99], *P* = 0.01), cases of female factor infertility (OR = 0.97, 95% CI [0.95–0.99], *P* = 0.01), cycles with a low Gn starting dose (OR = 0.97, 95% CI [0.94–1.00], *P* = 0.04), and those with single embryo transfer (OR = 0.98, 95% CI [0.96–1.00], *P* = 0.04). Furthermore, marginal associations were observed in women aged < 35 years (OR = 0.98, 95% CI [0.97–1.00], *P* = 0.06), men aged < 40 years (OR = 0.98, 95% CI [0.97–1.00], *P* = 0.06), and cycles using the antagonist protocol (OR = 0.98, 95% CI [0.97–1.00], *P* = 0.07) (Fig. 3). Notably, no significant effect modification by any subgroup variable was observed for the association between SCCD and clinical pregnancy or live birth (all *P* for interaction >0.05) (Figs. 2 and 3).

### Nonlinear association analysis of SCCD with clinical pregnancy and live birth

**Figure 4:**
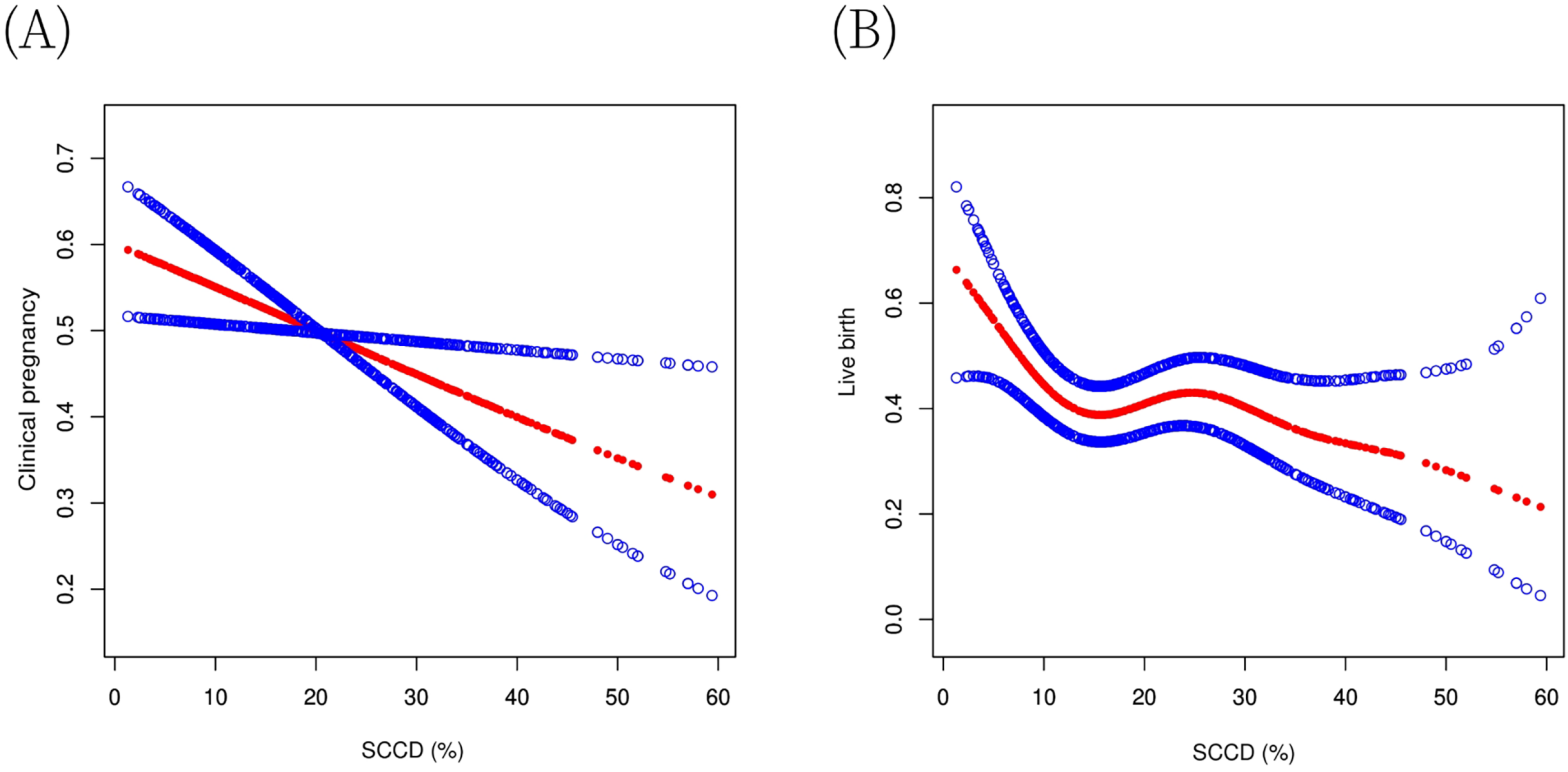
Non-linear associations between SCCD and pregnancy outcomes. Generalized additive model (GAM) curves showing the relationship between SCCD and (A) clinical pregnancy probability and (B) live birth probability. All models were adjusted for female age, male age, female BMI, infertility factors, AMH, baseline FSH, endometrial thickness, stimulation protocol, Gn starting dose, Gn duration, trigger day E2, embryos transferred, high-quality embryos transferred, and normal sperm morphology. Solid lines represent fitted values; shaded areas indicate 95% confidence intervals (CI). Abbreviations: SCCD, sperm chromatin condensation defect; GAM, generalized additive model; BMI, body mass index; AMH, anti-Müllerian hormone; FSH, follicle-stimulating hormone; Gn, gonadotropin; E2, estradiol; CI, confidence interval.

For clinical pregnancy, the association was linear. The linear regression model showed a significant negative association (adjusted OR = 0.98, 95% CI [0.96–1.00], *P* = 0.01). A piecewise regression model did not improve the fit (log-likelihood ratio test, *P* = 0.19) (Table 5), and the GAM plot visually supported this linear trend (Fig. 4A). In contrast, for live birth, a threshold effect was identified. The piecewise regression model with a turning point at 10.6% provided a significantly better fit than the linear model (log-likelihood ratio test, *P* = 0.02). Below this threshold (SCCD ≤ 10.6%), each 1% increase in SCCD was associated with a 14% decrease in the probability of live birth (adjusted OR = 0.86, 95% CI [0.77–0.97], *P* = 0.01). Above 10.6%, the association plateaued (adjusted OR = 0.99, 95% CI [0.97–1.01], *P* = 0.46) (Fig. 4B). Additionally, within the high SCCD population (>10.6%), we explored a potential secondary threshold. A secondary inflection point at 24.1% was suggested (log-likelihood ratio test, *P* = 0.06). Beyond this point (SCCD > 24.1%), a trend toward a further decline in live birth rates was observed (adjusted OR = 0.97, 95% CI [0.93–1.00], *P* = 0.07) (Table 5). Sensitivity analyses using simplified models supported these linear and non-linear patterns (Tables S2 and S3).

**Table 5:** Threshold effect analysis of the association between SCCD and clinical pregnancy and live birth outcomes.

	Clinical pregnancy (*n* = 618)	Live birth (*n* = 618)	Live birth (Subgroup: SCCD > 10.6, *n* = 501)
**Model 1: Linear model**	0.98 (0.96, 1.00) 0.01	0.98 (0.97, 1.00) 0.03	0.99 (0.97, 1.01) 0.57
**Model 2: Piecewise linear model**			
Inflection point (K), %	9.4	10.6	24.1
Segment 1 (SCCD < K)	0.89 (0.77, 1.03) 0.12	0.86 (0.77, 0.97) 0.01	1.04 (0.99, 1.10) 0.13
Segment 2 (SCCD > K)	0.99 (0.97, 1.00) 0.11	0.99 (0.97, 1.01) 0.46	0.97 (0.93, 1.00) 0.07
Ratio of ORs (Segment 2/Segment 1)	1.11 (0.95, 1.30) 0.19	1.15 (1.02, 1.31) 0.02	0.93 (0.86, 1.00) 0.06
Log(OR) at the inflection point	−0.00 (−0.27, 0.27)	−0.43 (−0.70, −0.16)	−0.15 (−0.48, 0.17)
Likelihood ratio test	0.19	0.02	0.06

**Note:**

Model 1 presents the linear effect. Model 2 presents the piecewise linear effect with an inflection point (K). Data are presented as OR (95% CI) *P*-value. All models were adjusted for female age, male age, female BMI, infertility factors, AMH, baseline FSH, endometrial thickness, stimulation protocol, Gn starting dose, Gn duration, trigger day E2, embryos transferred, high-quality embryos transferred, and normal sperm morphology. Abbreviations: SCCD, sperm chromatin condensation defect; OR, odds ratio; CI, confidence interval.

## Discussion

In 647 IVF cycles, SCCD was correlated with impaired semen parameters and a reduced 2PN cleavage rate. After multivariable adjustment, elevated SCCD was independently associated with lower clinical pregnancy and live birth rates, showing an approximate 35% reduction at the ≥30% threshold. A key finding was a non-linear relationship with live birth, characterized by an initial risk reduction below 10.6%, a plateau of stable risk until 24.1%, and a subsequent declining trend. Stratified analyses indicated no significant effect modification by any of the covariates tested.

Our finding that SCCD correlates with impaired semen parameters is consistent with prior observations in asthenoteratozoospermia (Hologlu et al., 2022). The observed reduction in normal fertilization (2PN) rates further aligns with previously reported associations between SCCD and suboptimal fertilization outcomes in IVF (Gu et al., 2009; Hammadeh et al., 1998). Mechanistically, defective chromatin packaging is postulated to hinder paternal pronucleus formation and subsequent zygotic genome activation, likely through impaired chromatin remodeling and aberrant epigenetic reprogramming (Huang et al., 2019; Smith et al., 2021; Zhang et al., 2024; Rahman et al., 2018). These cellular perturbations may manifest clinically as aberrant pronuclear dynamics and compromised embryonic development (Ribas-Maynou et al., 2023; Sheng et al., 2025). However, the literature remains inconsistent; for instance, Jumeau et al. (2022) reported that SCCD was associated with accelerated early embryonic cleavage, underscoring the complex and not fully elucidated nature of its impact. These discrepant results may be attributable to heterogeneity in study cohorts, methodologies for assessing chromatin, or other procedural differences.

This study yields two principal findings regarding SCCD in IVF outcomes. First, after multivariable adjustment, our data demonstrate a negative association between elevated SCCD and the probabilities of clinical pregnancy and live birth. This finding aligns with several previous reports (Gu et al., 2009; Ribas-Maynou et al., 2023), while contrasting with others (Angelopoulos et al., 1998; Wang et al., 2023). Second, our analysis further indicates that this relationship exhibits a non-linear component, specifically for live birth. When modelled as a continuous variable, SCCD displayed a complex relationship with live birth, characterized by a steep decline in live birth probability at levels below 10.6% and a suggested secondary inflection point at 24.1%, beyond which a further declining trend was observed. This pattern was corroborated by our categorical analysis, which showed a trend toward an approximate 35% reduction in the odds of live birth at the predefined SCCD ≥30% cutoff. This suggests that even minor impairments in chromatin packaging may be sufficient to disrupt crucial early embryonic events such as paternal epigenetic reprogramming, while a higher defect burden may exceed the oocyte’s reparative capacity, leading to embryonic developmental abnormalities and compromised outcomes (Jumeau et al., 2022; Marinaro, 2023; Nikolova et al., 2020). This concept of threshold-based effects is further supported by established cutoffs in other chromatin assessment methods, including the 25% aniline blue threshold for identifying poor quality semen (Hammadeh et al., 2001) and the 31% chromomycin A3 (CMA3) threshold for discriminating protamine deficiency and high oligoasthenoteratozoospermia (OAT) risk (Zandemami et al., 2012). Therefore, beyond differences in study populations and methodologies, the potential non-linear relationship may account for some of the heterogeneity in previous literature.

Stratified analysis substantiated the negative association between elevated SCCD and compromised IVF outcomes within several clinical subgroups. Notably, this consistency was observed not only across basic demographic and infertility-related categories but also across the different ovarian stimulation protocols used in this cohort (Figs. 2 and 3). Critically, the absence of significant effect modification demonstrates that this association remained statistically consistent across the wide spectrum of patient profiles examined. These findings suggest that the detrimental impact of SCCD may represent a pervasive factor in the IVF population, independent of specific patient characteristics.

This study has several limitations. First, its retrospective nature precludes causal inference. Second, the SCCD assessment, based on preoperative aniline blue staining, may not fully reflect the status on oocyte retrieval day and is subject to the inherent subjectivity of the method. Although the use of a fixed team of experienced technologists and a standardized protocol with duplicate counting mitigated analytical variation, the lack of a formal inter-observer assessment remains a constraint. Third, not all patients underwent pre-cycle SCCD testing, which may introduce selection bias and limit the generalizability of our findings. Finally, regarding statistical power, it is worth noting that the *post hoc* power for the dichotomized SCCD analysis (≥30% *vs*. <30%) for the primary outcomes in the multivariable models was 55–61%. This may be attributable to the inherent reduction of statistical information when converting continuous variables to categorical ones, which likely accounts for the observed borderline statistical significance (*P* = 0.05 and 0.06). However, the reliability of the findings remains robust, as our multivariable models maintained adequate EPV to ensure validity. Furthermore, the significant association observed when SCCD was analyzed as a continuous variable (*P* = 0.01–0.02), along with the consistent results from multiple sensitivity analyses (Table S1), confirms the robustness of our findings. Notably, cross-validation was omitted to preserve statistical power for detecting non-linear patterns. Consequently, the clinical utility of the identified thresholds warrants validation in larger, prospective cohorts.

## Conclusion

In conclusion, our findings indicate that elevated SCCD is associated with poorer clinical outcomes in IVF, supporting the potential clinical value of its assessment prior to treatment.

## Supplemental Information

10.7717/peerj.20749/supp-1 Supplemental Information 1 Sensitivity analyses of the association between SCCD and IVF outcomes using simplified adjustment models. **Note:** Associations were analyzed using multivariable logistic regression and are presented as OR with 95% CI. A two-sided *P* value of < 0.05 was considered statistically significant. Adjustd I: Female age, m ale age, female BMI, infertility factors. Adjustd II: Female age, Male age, female BMI, infertility factors, AMH, endometrial thickness, stimulation protocol, normal sperm morphology. **Abbreviations:** SCCD, sperm chromatin condensation def ects; IVF, *in vitro* fertilization; OR, odds ratio; CI, confidence interval; BMI, body mass index; AMH, Anti-Müllerian Hormone.

10.7717/peerj.20749/supp-2 Supplemental Information 2 Threshold e ffect a nalysis of SCCD on IVF o utcomes ( m inimally a djusted m odel). **Note: ** Model 1 presents the linear effect. Model 2 presents the piecewise linear effect with an inflection point (K). Data are presented as OR (95% CI) *P* -value. All models were adjusted for female age, male age, female BMI, infertility factors . **Abbreviations: ** SCCD, sperm chromatin condensation defects; OR, odds ratio; CI, confidence interval.

10.7717/peerj.20749/supp-3 Supplemental Information 3 Threshold e ffect a nalysis of SCCD on IVF o utcomes ( m ore c omprehensively a djusted m odel). **Note: Model 1** presents the linear effect. **Model 2** presents the piecewise linear effect with an inflection point (K). Data are presented as OR (95% CI) *P* -value. All models were adjusted for Female age, Male age, female BMI, infertility factors, AMH, endometrial thickness, stimulation protocol, normal sperm morphology. **Abbreviations:** SCCD, sperm chromatin condensation defects; OR, odds ratio; CI, confidence interval.

10.7717/peerj.20749/supp-4 Supplemental Information 4 Representative aniline blue staining of sperm chromatin condensation. (A) representative field from the normal SCCD group (<30%). (B) representative field from the high SCCD group (≥30%). According to the staining characteristics, spermatozoa with normal chromatin condensation (rich in protamines) appear reddish-pink, while those with abnormal condensation (containing lysine-rich histones) stain purplish-blue. Images were captured under oil immersion at 1000× magnification. Scale bar = 10 μm.

10.7717/peerj.20749/supp-5 Supplemental Information 5 Sensitivity analysis of the non-linear associations between SCCD and pregnancy outcomes using simplified adjustment models. Generalized additive model (GAM) curves showing the relationship between SCCD and (A, C) clinical pregnancy probability and (B, D) live birth probability. (A, B) Models adjusted for female age, male age, female BMI, and infertility factors. (C, D) Models adjusted for female age, male age, female BMI, infertility factors, AMH, endometrial thickness, stimulation protocol, and normal sperm morphology. Solid lines represent fitted values; shaded areas indicate 95% confidence intervals (CI). Abbreviations: SCCD, sperm chromatin condensation defects; CI, confidence interval.

10.7717/peerj.20749/supp-6 Supplemental Information 6 Raw data.

## Additional Information and Declarations

### Data Availability

The following information was supplied regarding data availability:

The raw data are available in the Supplemental File.
