# Supplementary material for: Sperm chromatin condensation defects and IVF outcomes: a retrospective cohort study
Source: PeerJ. 2026 Jan 29;14:e20749. doi: 10.7717/peerj.20749 (PMC12861134; doi:10.7717/peerj.20749)
Supplement: Supplemental Information 1 — Note: Associations were analyzed using multivariable logistic regression and are presented as OR with 95% CI. A two-sided P value of < 0.05 was considered statistically significant. Adjustd I: Female age, m ale age, female BMI, infertility factors. Adjustd II: Female age, Male age, female BMI, infertility factors, AMH, endometrial thickness, stimulation protocol, normal sperm morphology. Abbreviations: SCCD, sperm chromatin condensation def ects; IVF, in vitro fertilization; OR, odds ratio; CI, confidence interval; BMI, body mass index; AMH, Anti-Müllerian Hormone. [file peerj-14-20749-s001.docx]

|  | Adjusted Ⅰ | Adjusted Ⅱ |
| --- | --- | --- |
| **Clinical pregnancy** |  |  |
| Continuous | 0.99 (0.98, 1.00) 0.10 | 0.98 (0.96, 0.99) 0.01 |
| ＜ 30 | Reference | Reference |
| ≥ 30 | 0.80 (0.54, 1.19) 0.27 | 0.65 (0.43, 0.98) 0.04 |
| **Live birth** |  |  |
| Continuous | 0.98 (0.97, 1.00) 0.04 | 0.98 (0.97, 1.00) 0.01 |
| ＜ 30 | Reference | Reference |
| ≥ 30 | 0.75 (0.50, 1.13) 0.17 | 0.67 (0.44, 1.02) 0.06 |
