# Supplementary material for: Sperm chromatin condensation defects and IVF outcomes: a retrospective cohort study
Source: PeerJ. 2026 Jan 29;14:e20749. doi: 10.7717/peerj.20749 (PMC12861134; doi:10.7717/peerj.20749)
Supplement: Supplemental Information 2 — Note: Model 1 presents the linear effect. Model 2 presents the piecewise linear effect with an inflection point (K). Data are presented as OR (95% CI) P -value. All models were adjusted for female age, male age, female BMI, infertility factors . Abbreviations: SCCD, sperm chromatin condensation defects; OR, odds ratio; CI, confidence interval. [file peerj-14-20749-s002.docx]

|  | Clinical pregnancy  (n = 644) | Live birth  (n = 644) | Live birth  (Subgroup: SCCD > 9.4, n = 546) |
| --- | --- | --- | --- |
| **Model 1: Linear Model** | 0.99 (0.97, 1.00) 0.12 | 0.99 (0.97, 1.00) 0.08 | 1.00 (0.98, 1.01) 0.70 |
| **Model 2: Piecewise Linear Model** |  |  |  |
| Inflection point (K), % | 8.9 | 9.4 | 25.1 |
| Segment 1 (SCCD < K) | 0.88 (0.76, 1.02) 0.10 | 0.85 (0.74, 0.97) 0.01 | 1.03 (0.99, 1.07) 0.15 |
| Segment 2 (SCCD > K) | 0.99 (0.98, 1.01) 0.48 | 1.00 (0.98, 1.01) 0.65 | 0.97 (0.94, 1.00) 0.09 |
| Ratio of ORs (Segment 2 / Segment 1) | 1.12 (0.96, 1.31) 0.14 | 1.17 (1.02, 1.35) 0.024 | 0.94 (0.89, 1.01) 0.08 |
| Log(OR) at the inflection point | -0.05 (-0.31, 0.21) | -0.43 (-0.70, -0.17) | -0.19 (-0.51, 0.13) |
| Likelihood ratio test | 0.13 | 0.02 | 0.07 |
