# Supplementary material for: Sperm chromatin condensation defects and IVF outcomes: a retrospective cohort study
Source: PeerJ. 2026 Jan 29;14:e20749. doi: 10.7717/peerj.20749 (PMC12861134; doi:10.7717/peerj.20749)
Supplement: Supplemental Information 3 — Note: Model 1 presents the linear effect. Model 2 presents the piecewise linear effect with an inflection point (K). Data are presented as OR (95% CI) P -value. All models were adjusted for Female age, Male age, female BMI, infertility factors, AMH, endometrial thickness, stimulation protocol, normal sperm morphology. Abbreviations: SCCD, sperm chromatin condensation defects; OR, odds ratio; CI, confidence interval. [file peerj-14-20749-s003.docx]

|  | Clinical pregnancy  (n = 621) | Live birth  (n = 621) | Live birth  (Subgroup: SCCD > 10.3, n = 527) |
| --- | --- | --- | --- |
| **Model 1: Linear Model** | 0.98 (0.97, 1.00) 0.01 | 0.98 (0.97, 1.00) 0.02 | 0.99 (0.97, 1.01) 0.33 |
| **Model 2: Piecewise Linear Model** |  |  |  |
| Inflection point (K), % | 9 | 10.3 | 25 |
| Segment 1 (SCCD < K) | 0.91 (0.78, 1.06) 0.21 | 0.87 (0.78, 0.98) 0.02 | 1.02 (0.98, 1.07) 0.25 |
| Segment 2 (SCCD > K) | 0.98 (0.97, 1.00) 0.07 | 0.99 (0.97, 1.01) 0.34 | 0.97 (0.93, 1.00) 0.05 |
| Ratio of ORs (Segment 2 / Segment 1) | 1.08 (0.93, 1.27) 0.32 | 1.13 (1.00, 1.28) 0.05 | 0.94 (0.88, 1.01) 0.08 |
| Log(OR) at the inflection point | -0.00 (-0.27, 0.27) | -0.43 (-0.70, -0.16) | -0.17 (-0.50, 0.15) |
| Likelihood ratio test | 0.31 | 0.04 | 0.08 |
