# Supplementary figures and images for: Sperm chromatin condensation defects and IVF outcomes: a retrospective cohort study
Source: PeerJ. 2026 Jan 29;14:e20749. doi: 10.7717/peerj.20749 (PMC12861134; doi:10.7717/peerj.20749)

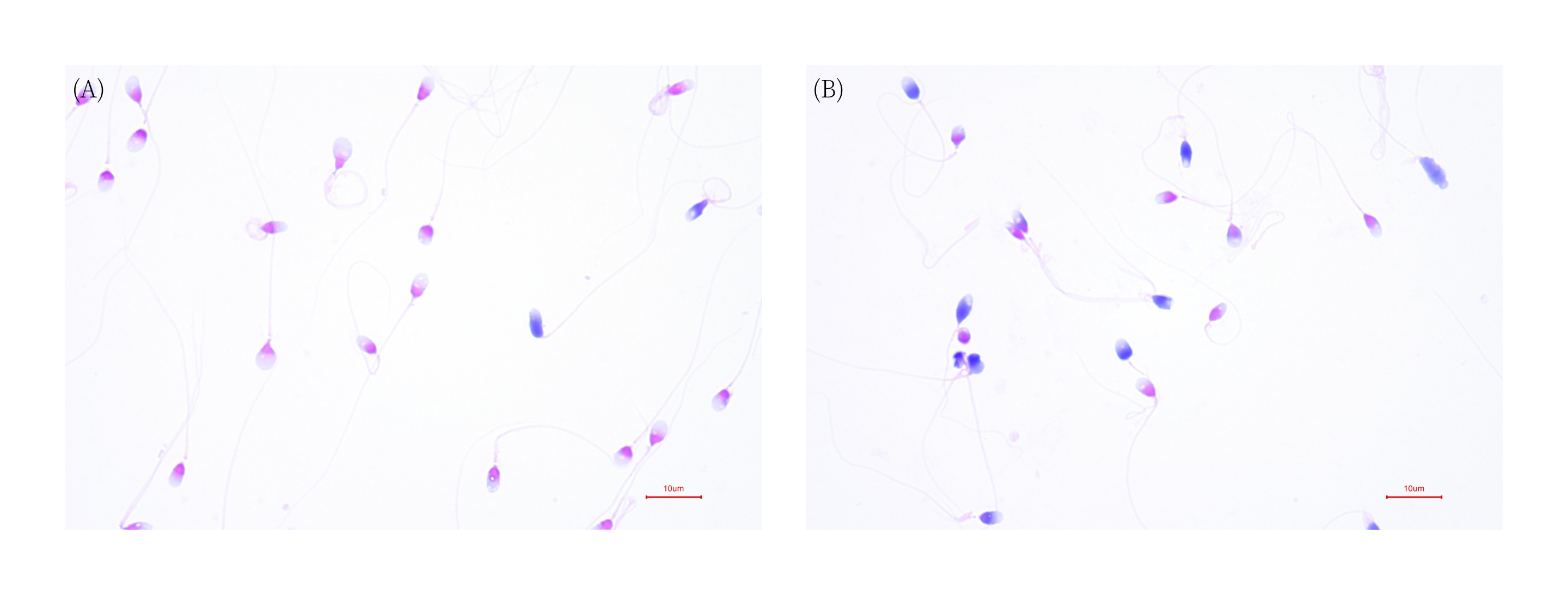

Supplement: Supplemental Information 4 — (A) representative field from the normal SCCD group (<30%). (B) representative field from the high SCCD group (≥30%). According to the staining characteristics, spermatozoa with normal chromatin condensation (rich in protamines) appear reddish-pink, while those with abnormal condensation (containing lysine-rich histones) stain purplish-blue. Images were captured under oil immersion at 1000× magnification. Scale bar = 10 μm. [file peerj-14-20749-s004.png]

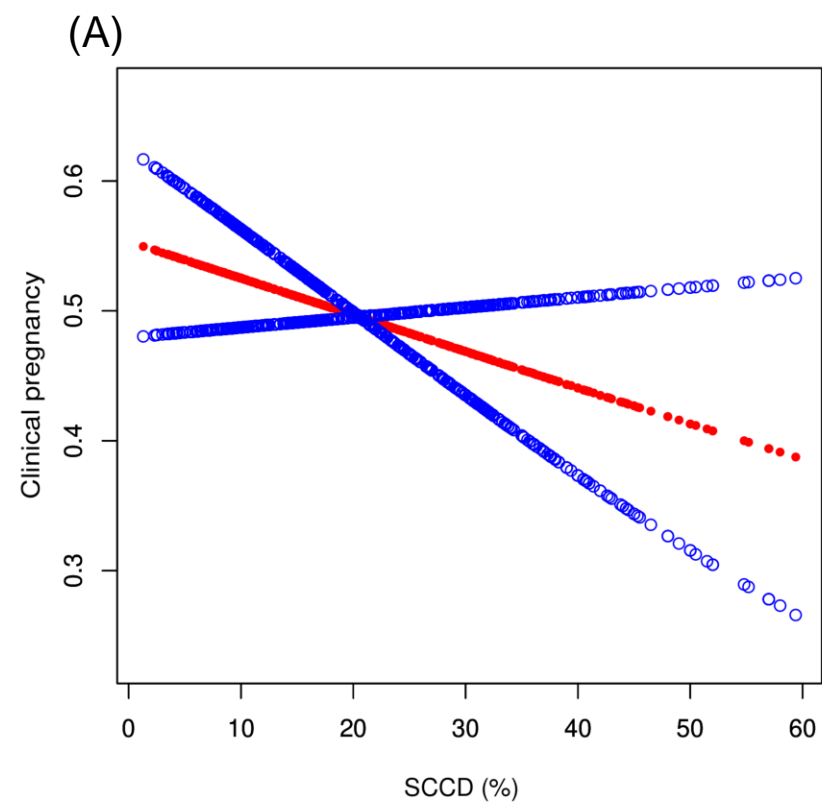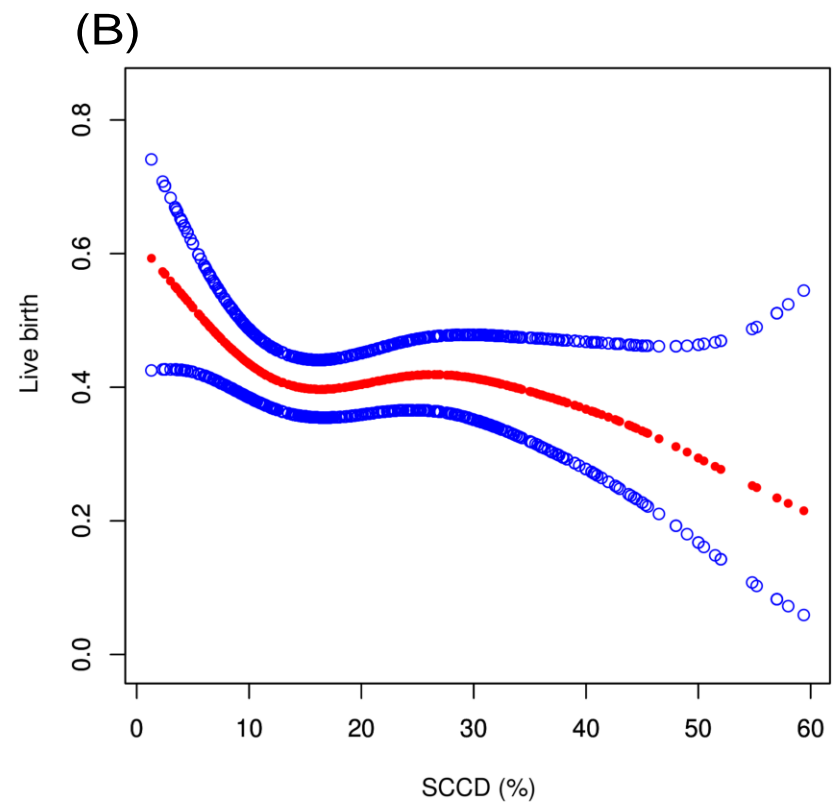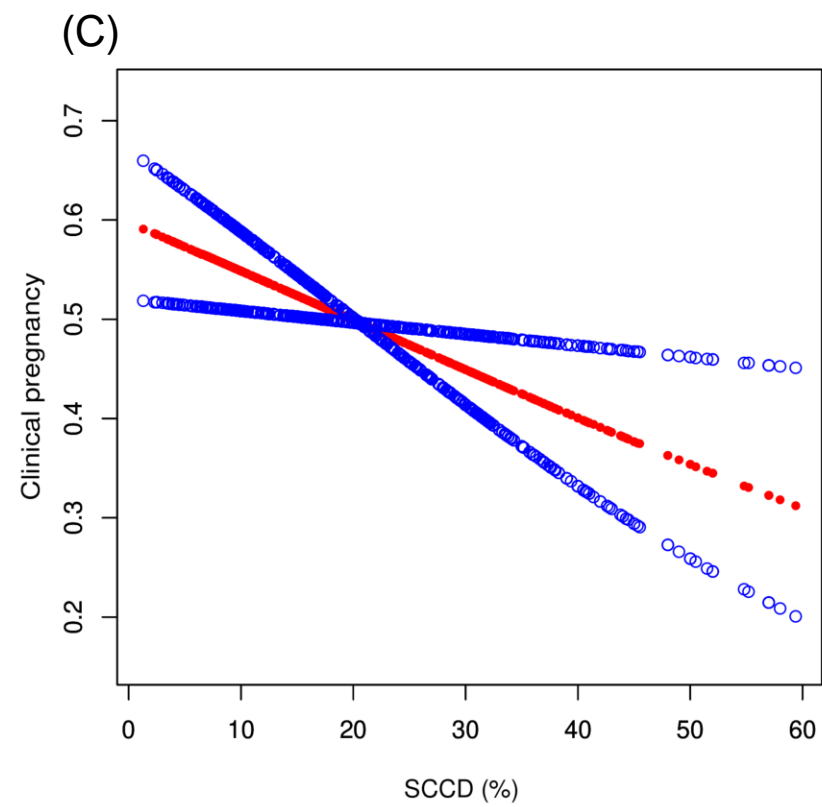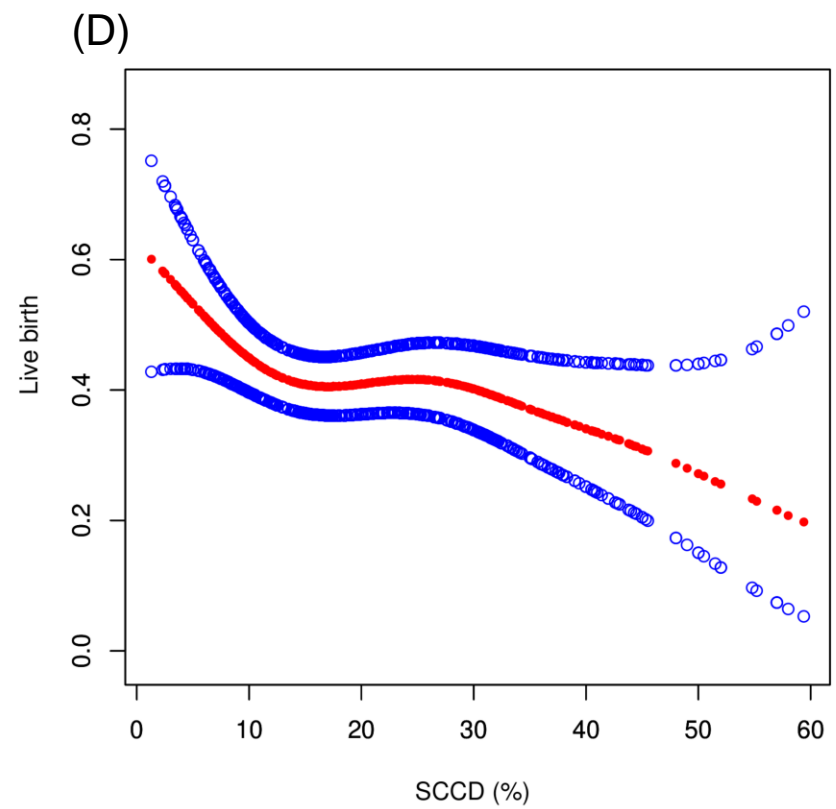

Supplement: Supplemental Information 5 — Generalized additive model (GAM) curves showing the relationship between SCCD and (A, C) clinical pregnancy probability and (B, D) live birth probability. (A, B) Models adjusted for female age, male age, female BMI, and infertility factors. (C, D) Models adjusted for female age, male age, female BMI, infertility factors, AMH, endometrial thickness, stimulation protocol, and normal sperm morphology. Solid lines represent fitted values; shaded areas indicate 95% confidence intervals (CI). Abbreviations: SCCD, sperm chromatin condensation defects; CI, confidence interval. [file peerj-14-20749-s005.pdf]
